# The Stigma of Mental Healthcare: A Qualitative Analysis of the Utilisation of Mental Health Services by South Asians in the UK

**DOI:** 10.1192/j.eurpsy.2023.1735

**Published:** 2023-07-19

**Authors:** J. Shukla

**Affiliations:** School of Art, Languages and Culture, University of Manchester, Manchester, United Kingdom

## Abstract

**Introduction:**

A growing amount of evidence indicates that South Asians in the UK are experiencing high rates of mental health disorders. Despite this, data from the National Health Service reveals that these communities utilise mental health services less than any other ethnic group residing in the UK. For these communities, mental illness stigma has been cited as a major barrier to accessing mental healthcare.

**Objectives:**

By situating stigma within a specific socio-cultural context, this study aimed to explore how stigma may affect the utilisation of mental healthcare by South Asian communities within the UK. Acknowledging that the experiences of stigma can be influenced by an intersection of multiple social categorisations, it aimed to disaggregate the data further. This study examined how the experiences of stigma and the subsequent utilisation of mental healthcare may differ across generational statuses.

**Methods:**

This qualitative study utilised document analysis as its approach to data collection and interpretation. Both, academic and grey literature were used. A literature search was performed using Google Scholar and PubMed. Google search engine was also used to identify grey literature such as blogs and reports. The search terms “mental health services”, “stigma”, “utilisation”, “South Asian”, “culture” and “generation” were used. Articles were organised into matrices relating to cultural stigma and intersections relating to culture and generational status.

**Results:**

This study revealed that the values and beliefs instilled within South Asian cultures may perpetuate the effects of stigma, thus contributing to the under-utilisation of mental health services within the UK. This study also observed that second-generation South Asians may be more likely to utilise mental health services in comparison to the first-generation. This is ascribed to the fact that second-generation individuals, as a result of processes related to acculturation, are more likely to disengage with their heritage culture, which may, in turn, mitigate the levels of stigma experienced and reduce barriers to care.

**Conclusions:**

This research argues that stigma is socially constructed within cultural groups, and heterogeneously across generations. Such findings can have wider relevance for healthcare professionals, who wish to become more culturally competent and policymakers, who wish to tailor anti-stigma interventions. Primary qualitative research, using questionnaires and interviews, should be employed to further understand mental illness stigma within cultural groups, such as South Asians, and could prove useful in acknowledging discrete beliefs between first- and second-generation cultural groups.

**Disclosure of Interest:**

None Declared

